# Optogenetic control of YAP can enhance the rate of wound healing

**DOI:** 10.1186/s11658-023-00446-9

**Published:** 2023-05-11

**Authors:** Pearlyn Jia Ying Toh, Marius Sudol, Timothy Edward Saunders

**Affiliations:** 1 Mechanobiology Institute, National University of Singapore, Singapore, Singapore; 2 Faculty of Health and Medical Sciences, Novo Nordisk Foundation Center for Basic Metabolic Research, University of Copenhagen, Copenhagen, Denmark; 3 Icahn School of Medicine at Mount Sinai, New York, NY USA; 4 Institute of Molecular and Cell Biology, A*STAR, Singapore, Singapore; 5 Warwick Medical School, University of Warwick, Coventry, UK

**Keywords:** Hippo-YAP, Optogenetics, Wound healing

## Abstract

**Background:**

Tissues need to regenerate to restore function after injury. Yet, this regenerative capacity varies significantly between organs and between species. For example, in the heart, some species retain full regenerative capacity throughout their lifespan but human cardiac cells display a limited ability to repair the injury. After a myocardial infarction, the function of cardiomyocytes is impaired and reduces the ability of the heart to pump, causing heart failure. Therefore, there is a need to restore the function of an injured heart post myocardial infarction. We investigate in cell culture the role of the Yes-associated protein (YAP), a transcriptional co-regulator with a pivotal role in growth, in driving repair after injury.

**Methods:**

We express optogenetic YAP (optoYAP) in three different cell lines. We characterised the behaviour and function of optoYAP using fluorescence imaging and quantitative real-time PCR of downstream YAP target genes. Mutant constructs were generated using site-directed mutagenesis. Nuclear localised optoYAP was functionally tested using wound healing assay.

**Results:**

Utilising optoYAP, which enables precise control of pathway activation, we show that YAP induces the expression of downstream genes involved in proliferation and migration. optoYAP can increase the speed of wound healing in H9c2 cardiomyoblasts. Interestingly, this is not driven by an increase in proliferation, but by collective cell migration. We subsequently dissect specific phosphorylation sites in YAP to identify the molecular driver of accelerated healing.

**Conclusions:**

This study shows that optogenetic YAP is functional in H9c2 cardiomyoblasts and its controlled activation can potentially enhance wound healing in a range of conditions.

**Supplementary Information:**

The online version contains supplementary material available at 10.1186/s11658-023-00446-9.

## Introduction

During myocardial infarction, blood flow to the heart is severely limited or completely cut off by plaque or cholesterol blockages. Without oxygen and nutrients, heart muscles surrounding the region of the blocked vessel undergo necrosis. Acute myocardial injury can kill 25% of cardiomyocytes, rendering them non-functional [1]. Pharmacological management post myocardial infarction only serves to prevent another episode from occurring and there are no known strategies to restore the full capacity of the heart. This is in part due to the human heart having a very limited ability to regenerate post injury [2] and the lack of regenerative capability contrasts with other organs, such as the liver or gut [3]. However, other organisms show a greater capacity to regenerate injured heart tissue post-embryonic development: young mice (< 7 days) can repair heart wounding; and in zebrafish the potential to regenerate heart tissue remains throughout the lifespan [4, 5]. Therefore, there is a demand to find strategies to induce cardiac regeneration in humans. Though new cardiomyocytes can be synthesised ex vivo from embryonic stem cells and organise to form contractile tissues, injected cells have an embryonic lineage and can generate arrythmias [6, 7]. Recipients also need to be immunosuppressed for transfusion, which itself is a complex procedure. Alternative strategies to induce human heart tissue repair and regeneration are still heavily sought after.

A strategy to circumvent the above complexity is to stimulate the endogenous proliferation potential of cells to repair. The key idea is to mimic developmental processes, reviving pathways that are switched off postnatally. This approach has seen positive results in transgenic mice models, where activated Yes-associated protein (YAP) promotes cardiac regeneration by inducing reparative genetic programs to re-enter the cell cycle and activate proliferative genes in cardiomyocytes [8–10]. YAP is a central effector in the Hippo-YAP pathway, which is highly implicated in development and regeneration due to its role in driving cell proliferation, migration and mechanosensing [11–13]. The nucleocytoplasmic distribution of YAP is a key determinant of its activity. Therefore, manipulating the YAP subcellular localisation can provide a tool to control the activity of the Hippo-YAP pathway.

During regeneration, there are two main mechanisms for repair: (i) migration of newly proliferated cells into the injured area; and (ii) cell proliferation within the regenerating zone. YAP is involved in wound healing by limiting cytoskeletal and focal adhesion maturation to promote cell motility [14]. YAP increases migration and invasiveness in a number of cell lines, often stimulating epithelial-to-mesenchymal transition (EMT) [15]. On top of enhancing cell cycle progression, YAP also promotes cytoskeletal remodelling, responding to the mechanical change during scar tissue formation post myocardial infarction [16]. YAP has also been shown to be activated in cardiac fibroblasts after ischemic injury [17].

While YAP activity has been shown to regenerate the mice heart after injury, caution must be taken as over-proliferation can cause occlusive vascular diseases [18]. Therefore, having control over the spatiotemporal localisation of YAP is important. Optogenetics provides such a method [19, 20], whereby light activation can control protein behaviour, and we have recently applied this to YAP, generating an optogenetic YAP construct termed optoYAP [21]. Can optogenetic YAP be used to control regeneration after wounding or injury? optoYAP is potentially a powerful tool to circumvent regenerative limitations whilst retaining specific spatiotemporal control over its activity. Building upon our previously reported work [21], we utilise a range of cell lines, including H9c2 rat cardiomyoblasts, as in vitro models to study how activation of YAP can promote repair post-injury.

Here, we report that activated optoYAP accelerates wound healing in two different cell lines, driving cell migration that is essential during regeneration. In particular, we show that H9c2 cardiomyoblasts are responsive to optoYAP activation, inducing nuclear accumulation after light activation. Nuclear optoYAP increases expression of downstream proliferative YAP target genes *CTGF* and *CYR61*, as well as *TGF-β*, a transforming growth factor involved in stem cell regulation and differentiation. optoYAP also promotes wound healing in H9c2 and MKN28 cells through cell migration but not proliferation. This suggests that optoYAP can potentially be applicable in regenerative studies of tissues—including cardiac tissues—by promoting the renewal and replication of cells post-injury. Additionally, we observed that mutant optoYAP constructs that do not typically localise in the nucleus can be forced to do so by optogenetic activation. Overall, our results reveal that optogenetic approaches provide a strategy for specific control over YAP nuclear localisation (and hence activity) in both wildtype YAP and its mutants, thereby making it a potential tool for driving tissue regeneration.

## Materials and methods

### Plasmid construction

Optogenetic plasmid containing YAP (optoYAP) was previously described by Toh et al., 2022 [21]. Mutant YAP containing S127A or ΔC modifications were PCR amplified from pBABE (hygro) hYAP1-1δ S127A and pBABE (hygro) hYAP1-1δ ΔC plasmids respectively, both of which were from the library of YAP complementary DNA (cDNA) constructs of the Sudol laboratory. These constructs are available from ‘Addgene’ vector resource. Phusion High Fidelity DNA polymerase (ThermoScientific) was used for PCR amplification with primer sequences for each amplification listed in Table 1. PCR products and optoYAP plasmid were cleaved with appropriate FastDigest restriction enzymes (ThermoScientific) for cloning.

**Table 1:** List of primers for plasmid cloning

Primer name	Sequence	Cloning	Restriction enzyme
PT25 hYAP1-1delta fwd	ATTTATGGTACC ATGGATCCCGGGCAG	Forward primer for S127A and ΔC mutants	KpnI
PT26 hYAP1-1delta S127A rev	CGCGCCGTTTAAACC TATAACCATGTAAG	Reverse primer for S127A	PmeI
PT27 hYAP1-1delta DeltaC rev	CGCGCCGTTTAAACC TAGCTTTCTTTATCTAGC	Reverse primer for ΔC	PmeI
795-2194-YAP-S251D-F1	GATCCACAGGGAGG CGTCATGGGTG	Forward primer for SDM of S251D mutant	–
796-2194-YAP-S251D-R1	CTGGGGAGCCAGGG GTGGTGGCTGT	Reverse primer for SDM of S251D mutant	–
797-2195-YAP-S333D-F1	GATCCCGGGATGTC TCAGGAATTGAG	Forward primer for SDM of S333D mutant	–
798-2195-YAP-S333D-R1	CGACACTGGATTTT GAGTCCCACCATC	Reverse primer for SDM of S333D mutant	–
799-2196-YAP-2mut-F1	CAGCCACCACCCCTGGC TCCCCAGGATCCACAGG GAGGCGTCATGGGTG	Forward primer for SDM of S251D and S333D mutant	–
800-2196-YAP-2mut-R1	CTCAATTCCTGAGACATC CCGGGATCCGACACTGG ATTTTGAGTCCCACCATC	Reverse primer for SDM of S251D and S333D mutant	–

Point mutations in hYAP1-1δ were generated with Q5 Site-Directed Mutagenesis Kit (NEB) according to the manufacturer’s instructions to synthesise phosphomimic mutants by substituting serine with aspartic acid at two residues, S251 and S333. These two serine residues in the sequence of the hYAP1-1δ isoform correspond to S274 and S352, respectively, in mYAP1-2α, described by Aharonov and colleagues [22]. The differences in the splicing isoforms of hYAP1-1δ and mYAP1-2α are illustrated in Additional file 1 and 2. Positive clones were verified by restriction enzyme digestion and sequencing. Primers used for site-directed mutagenesis (SDM) are listed in Table 1.

### Mammalian cell culture and transfection

HEK293T (ATCC CRL-3216) human embryonic kidney cells were grown in Dulbecco’s modified Eagle’s medium (DMEM, ThermoScientific) supplemented with 10% (v/v) heat inactivated fetal bovine serum (FBS, Gibco) and 1% (v/v) penicillin/streptomycin (Gibco). MKN28 (CVCL_1416) human gastric adenocarcinoma cells were grown in RPMI (ThermoScientific) supplemented with 10% (v/v) FBS and 1% (v/v) penicillin/streptomycin. H9c2 (ATCC CRL-1446) rat cardiomyocytes (a gift from Dr Manvendra K. Singh’s lab) were grown in DMEM supplemented with 15% (v/v) FBS, 1% (v/v) penicillin/streptomycin and 1% non-essential amino acids (NEAA, Gibco). All cell cultures were kept in a 37 ºC incubator with 5% CO_2_ and passaged every 2–3 days. Cells were routinely checked for mycoplasma contamination and short tandem repeat (STR) profiled (1st Base).

HEK293T cells were transfected with plasmid DNA using Lipofectamine 2000 (ThermoScientific) using 6 µL of Lipofectamine 2000 reagent in 150 µL opti-MEM (ThermoScientific) and 3.5 µg of plasmid DNA in 175 µL opti-MEM according to the manufacturer’s instructions. H9c2 cells were transfected with Lipofectamine 2000 as per HEK293T cells, but with 5 µg of plasmid DNA. MKN28 cells were transfected using K2 transfection system (Biontex) according to the manufacturer’s instructions.

### Fluorescence microscopy

Live imaging was performed on Perkin Elmer spinning disk with a LUCPlanFLN 40×/0.6 numerical aperture (NA) air objective or Nikon Ti-E with Yokogawa W1 spinning disk with a PLAN APO VC 60×/1.20 NA water immersion objective. Cells were visualised under the microscope 24 h post transfection in a 37 ºC heated and humidified chamber.

Long term imaging of wound healing experiments was performed on widefield Nikon Biostation IMQ with 10 × air objective or Olympus IX81 with a UPLFLN 10×/0.3 NA air objective. The samples were tracked every 30 min to measure the rate of wound closure until the gap was completely closed.

### Image analysis

The images were analysed with Fiji ImageJ software. The nuclear to cytoplasmic ratio was measured by drawing a region of interest (ROI) around the cell as well as its nucleus. Unactivated optoYAP is excluded from the nucleus. Therefore, the region without mCherry signal before the start of the activation protocol was visually identified as the nucleus during quantification. A macro plugin written by Richard De Mets was used to mask the nucleus from the cytoplasm to calculate the mean intensity in the nucleus and cytoplasm based on the ROI drawn. The ROI was drawn for each cell analysed at every time frame. The wound healing analysis was performed using a macro written by Ong Hui Ting, which measures the changes in gap area of the wound over time. Charts were made with Prism 8 software. All figures were assembled with Adobe Illustrator.

### Light activation

Pulsed light activation followed a protocol of 1 s pulse of 0.15 mW 488 nm laser light followed by a 30 s dark phase. This pulsation is continued for 20 min, which constitutes as ‘light activation’. During recovery, the entire set up is kept in the dark for a further 20 min. Throughout the entire 40 min of light activation and recovery, imaging was done with a 561 nm laser line to image the mCherry fluorescent protein. For samples kept under dark conditions, aluminium foil was used to wrap the sample to prevent light contamination.

### Quantitative real-time PCR (RT-qPCR)

Transfected cells were subjected to 48 h of pulsatile light activation and total RNA was extracted from cells using RNeasy Plus Mini Kit (QIAGEN) and QIAshredder (QIAGEN) according to the manufacturer’s instructions. RNA was eluted with 40 µL nuclease-free water (NFW) and quantitated using NanoDrop 2000 spectrophotometer. cDNA was synthesised using SuperScript IV Reverse Transcriptase (ThermoScientific) using oligo d(T) primers according to the manufacturer’s instructions.

Real-time PCR detection were done using SYBR Green PCR Master Mix for 40 cycles in a Bio-Rad CFX96 thermal cycler. The threshold cycle (Ct) value for each gene was normalised to the Ct value of a housekeeping gene, *EIF1B*. The relative fold changes were calculated using ΔΔCt method. The primer sequences for target genes are listed in Table 2.

**Table 2:** Primers used for RT-qPCR

Target gene	Forward primer	Reverse primer
*EIF1B*	TGATGCAACT AAGGGTGACG	CAGTGTCTTT CTGCCGTTCC
*CTGF*	TGCATCCGTA CTCCCAAAAT	ATGTCTTCAT GCTGGTGCAG
*CYR61*	TCCCTGTTTT TGGAATGGAG	GAGCACTGGG ACCATGAAGT
*TGF-β*	TACCTGAACCC GTGTTGCTCTC	GTTGCTGAGGT ATCGCCAGGAA

### Wound healing assay

Cells were grown in their respective serum free media for 24 h before 25,000 cells per chamber were plated into Culture-Insert 2 well in µ-Dish 35 mm, high (ibidi). Cells were kept in serum free media throughout the experiment. 24 h after seeding, or when cells reach confluency in the chambers, the culture insert was removed with clean forceps under sterile conditions and the dish was filled with 2 mL of serum free media for imaging. Proliferating cells were defined as rounded cell doublets observed during imaging. These cell doublets were tracked and counted throughout the timelapse. An example of a proliferating cell doublet is highlighted in red in Fig. 2A.

### Statistical analysis

Biological replicates refer to independent experimental replicates done on different days with fresh biological samples and independent transfections and measurements. Technical replicates refer to repeated measurements of the same sample that represent independent measures of the random noise associated with protocols or equipment [23]. For comparisons between the same sample in different light and dark conditions, a paired t-test was used. For comparisons between samples with more than two conditions or groups, a two-way ANOVA test was used. For small sample sizes, estimation statistics was used to show the magnitude of the testing condition [24].

### Curve fitting

Curve fitting for the import and export rates was performed in MATLAB using the curve fitting function *fit*. Time constant (τ) represents the time scale over which the signal changes before or after light activation. Nuclear import rate (τ_a_) was fitted with $f\left(x\right)=a+b\left(1-{e}^{- \frac{x}{\tau }}\right)$ and export rate (τ_r_) was fitted with $f\left(x\right)=a+b\left({e}^{- \frac{x}{\tau }}\right)$, where *a* is the basal level of signal intensity; and *b* is the multiplicative factor representing the change in signal due to light (de-)activation.

## Results

### optoYAP activity in H9c2 cells

**Fig. 1:**
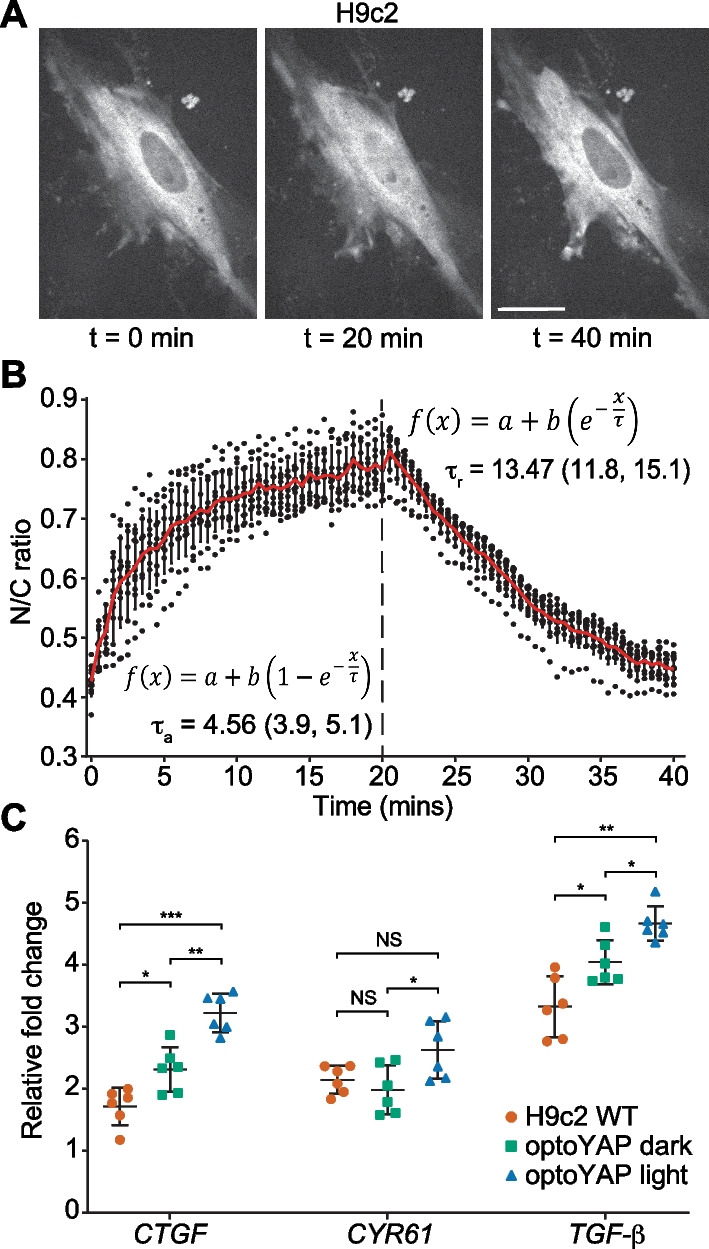
Characterisation of optoYAP in H9c2 cells. **A** Representative images of H9c2 cells transfected with optoYAP kept in the dark (t = 0 min), then subjected to pulsatile light activation for 20 min (t = 20 min), followed by recovery in the dark for a further 20 min (t = 40 min). Scale bar, 20 µm. **B** Quantification of activation (τ_a_) and recovery (τ_r_) time constant of optoYAP. Red line represents average nuclear/cytoplasmic ratio. Vertical dashed line represents time when 488 nm stimulation ceased. Numbers in brackets represent the 95% confidence interval of τ. n = 12 cells. **(C)** RT-qPCR of YAP downstream genes. Cells were transfected with optoYAP, and transcript levels were assessed after 48 h of activation. Expression levels of *CTGF*, *CYR61*, and *TGF-β* were normalised to the housekeeping gene *EIF1B* and described as relative fold change. Horizontal bars represent mean and 95% confidence interval from 6 biological replicates from two independent experiments. *NS* not significant, *P < 0.05, **P < 10^–2^, ***P < 10^–3^

Using a previously reported optoYAP for optogenetic control of YAP nuclear import and export [21], we transfected H9c2 cells with optoYAP and characterised the construct in rat cardiomyoblasts. We subjected transfected cells to pulsatile light activation with 488 nm laser power at 0.15 mW for 1 s at a 30 s interval, for a total of 20 min. We observed nuclear localisation of the optogenetic construct after the 20 min activation window (Fig. 1A). After leaving the cells in the dark for a further 20 min, optoYAP translocated out of the nucleus and returned to the basal state before light activation (Fig. 1A). The nuclear/cytoplasmic ratio of optoYAP almost doubled after the 20 min activation (Fig. 1B). Throughout the activation and recovery cycle, we tracked and imaged the cells over time to obtain the nuclear import and export dynamics (Fig. 1B). The activation (τ_a_) and recovery (τ_r_) time constants were calculated to be 4.6 ± 0.6 min and 13.5 ± 1.6 min respectively ("Methods" section). These values are comparable with those obtained by using the HEK293T cell line described previously, where it has been thoroughly characterised [21].

### Elevated downstream gene expression levels after optoYAP activation

Following the light-dependent nuclear localisation of optoYAP, we examined the functionality of nuclear localised optoYAP by measuring the changes in downstream gene expression levels. We quantified the transcript levels of *CTGF*, *CYR61*, and *TGF-β* in H9c2 cells after 48 h of pulsatile light activation (Fig. 1C). *CTGF* and *CYR61* are canonical YAP target genes that provide a readout for optoYAP activity in the nucleus [25, 26]. Additionally, we looked at *TGF-β* transcript levels, an inflammation-related growth factor that is frequently involved in the migration and invasion of tumour cells [27]. YAP has also been shown to be a mediator of TGF-β signalling [28, 29] and the pathway has been implicated in cardiomyocyte proliferation and migration [30, 31].

We observed that the expression levels of all three genes probed were significantly upregulated in cells containing activated optoYAP as compared to wildtype (WT) H9c2 (Fig. 1C). However, the transcript levels of *CTGF* and *TGF-β* in transfected cells kept in the dark were also significantly increased in comparison to wildtype controls, though clearly less than when light activated. This suggests that there are small amounts of nuclear optoYAP even without light activation. However, the upregulation of gene expression across the three genes is significant when comparing cells kept in the light versus dark. This shows that the optoYAP construct is functional when nuclear localised and can trigger expression of downstream target genes.

### optoYAP promotes collective cell migration

**Fig. 2:**
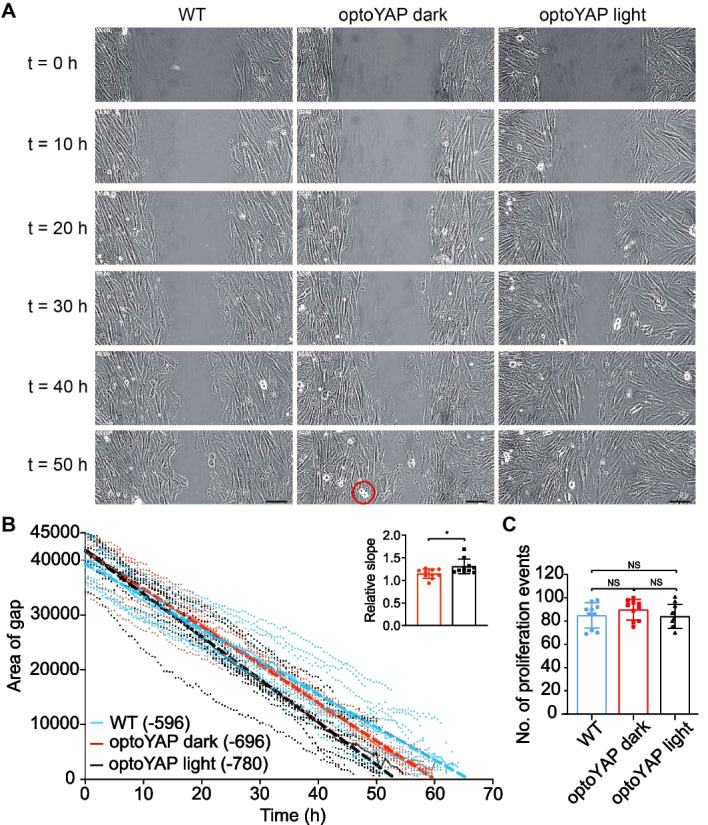
Wound healing assay in H9c2 cells.** A** Timelapse images of wildtype H9c2 cells or cells transfected with optoYAP under different light conditions, taken every 30 min until gap completely closes. Representative images shown at 10 h intervals. Rounded cell doublet circled in red (bottom middle panel) is an example of a proliferating event. Scale bars, 100 µm. **B** Time taken for H9c2 cells for complete wound healing. Quantification of data in **A** over time. Bold lines represent mean and dashed lines represent linear regression fitted to the curve. n = 10 from two independent experiments. Numbers in brackets represent gradient of linear regression slope. Inset shows the measured slopes under different conditions relative to wildtype (colours as in main plot), error bars are s.d.. **C** Number of proliferation events counted during each wound healing experiment in (B). Bars represent mean and error bars represents s.d.. *P < 0.05, *NS* not significant

We next investigated the role of optoYAP during wound healing, comparing endogenous and optogenetic YAP. Serum-starved cells were seeded in wells 500 µm apart in serum-free media and the "wound” size was quantified every 30 min until complete closure. We observed that optoYAP transfected H9c2 cells kept under pulsatile light activation migrated faster over the wound as compared to transfected cells kept in the dark or untransfected H9c2 (Fig. 2A). Wildtype H9c2 cells took an average of 66 h for complete wound closure, in comparison to 60 h taken for transfected cells in the dark (Fig. 2B). Light activated optoYAP accelerated wound healing, taking an average of 53 h, a 20% improvement on the wildtype cells. Though optoYAP transfected H9c2 cells kept in the dark took a shorter time as compared to wildtype cells, light activation of the optogenetic construct clearly bolstered this behaviour. Extracting the curve slopes for individual experiments in Fig. 2B, we see that the activated optoYAP cells have an increased rate of closure (Fig. 2B inset).

We observed some cell proliferation events during the timelapse, despite the serum starvation before and during the experiment, seen as bright, rounded cell doublets in Fig. 2A. We counted the number of proliferation events that occurred during wound healing and observed that all three conditions had similar number of proliferation events (Fig. 2C). This shows that the differences in the time taken for wound healing observed in Fig. 2B is largely due to cell migration and not proliferation.

We performed the same wound healing experiment in another cell line, MKN28, to exploit the benefit of a CRISPR *YAP*^*−/−*^ background previously reported [32]. This cell line enabled us to precisely delineate the effect of optoYAP without any endogenous YAP present that might confound the above results. Previous studies in the same cell line observed a difference between wildtype and *YAP*^*−/−*^ cells in a trans-well migration assay but not during wound healing assays [32]. Therefore, we wanted to determine if our optoYAP construct changes the rate of wound closure in these MKN28 *YAP*^*−/−*^ cells.

**Fig. 3:**
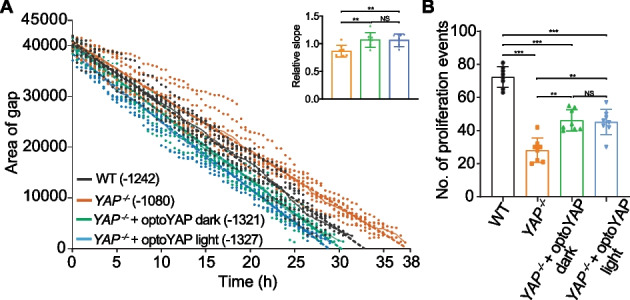
Wound healing assay in MKN28 variants.** A** Wildtype (WT) MKN28 and MKN28 *YAP*^*−/−*^ cells transfected with optoYAP were seeded in culture wells 500 µm apart. The size of the gap was tracked every 30 min until it was completely closed. Bold lines represent mean and dashed line represents linear regression fitted to curve. n = 8 from two independent experiments. Numbers in brackets represent gradient of linear regression slope. Inset shows slope of lines relative to wildtype (colours as in main plot), with error bars representing s.d.. **B** Number of proliferation events in each experiment in (**A**). Bars represent mean and error bars represents s.d.. **P < 10^–2^, ***P < 10^–3^, *NS* not significant

As expected, the *YAP*^*−/−*^ cells took much longer than the wildtype or optoYAP transfected cells for wound closure, taking 38 h as compared to 33 h for wildtype MKN28 cells (Fig. 3A). This shows that endogenous YAP is required for cell motility during wound closure. In MKN28 *YAP*^*−/−*^ cells transfected with optoYAP, we noticed that those kept in the dark took a faster time for wound closure (30.5 h) in comparison to wildtype cells. Light activated optoYAP transfected *YAP*^*−/−*^ cells had an accelerated rate of wound closure, taking only 29 h for the wound to close completely. This is supported by analysis of the slope of the curves (Fig. 3A inset). These results support the conclusion that endogenous YAP is involved in cell migration and optoYAP further accelerates wound healing in MKN28 cells. Globally, it was noteworthy that the MKN28 cells took a shorter time for wound closure (< 40 h) as compared to the H9c2 cells (> 50 h).

As with the H9c2 cells, we observed several proliferating MKN28 cells in the cell islands during wound closure. We counted the number of proliferation events and compared it amongst the MKN28 variants (Fig. 3B). Unsurprisingly, the *YAP*^*−/−*^ variants had the least amount of proliferation events (28 ± 7), less than half of the wildtype cells (72 ± 6) over the entire time course. Both optoYAP transfected *YAP*^*−/−*^ cells under light (45 ± 7) or dark (46 ± 6) conditions had similar number of proliferation events, but still less than wildtype cells. Despite less proliferation observed in optoYAP transfected *YAP*^*−/−*^ cells, the time taken for wound closure is shorter than wildtype cells. This shows that optoYAP speeds up the rate of wound closure in *YAP*^*−/−*^ cells, seemingly by inducing cell migration as proliferation levels were not rescued to wildtype states but wound closure took a shorter time in optoYAP-transfected cells. However, cell proliferation plays a crucial part in wound closure, and it is important to understand the fine interplay between proliferation and migration as revealed here by using CRISPR/Cas9-generated *YAP*^*−/−*^ MKN28 cells.

### Changes in nuclear localisation of optoYAP mutants

Above, through our careful quantitative analysis, we have clearly shown that YAP can play a role in wound healing, including in cardiomyocytes. Next, we looked to understand in further detail the elements of YAP – *e.g.,* phosphorylation—that are acting to increase the rate of wound healing.

**Fig. 4:**
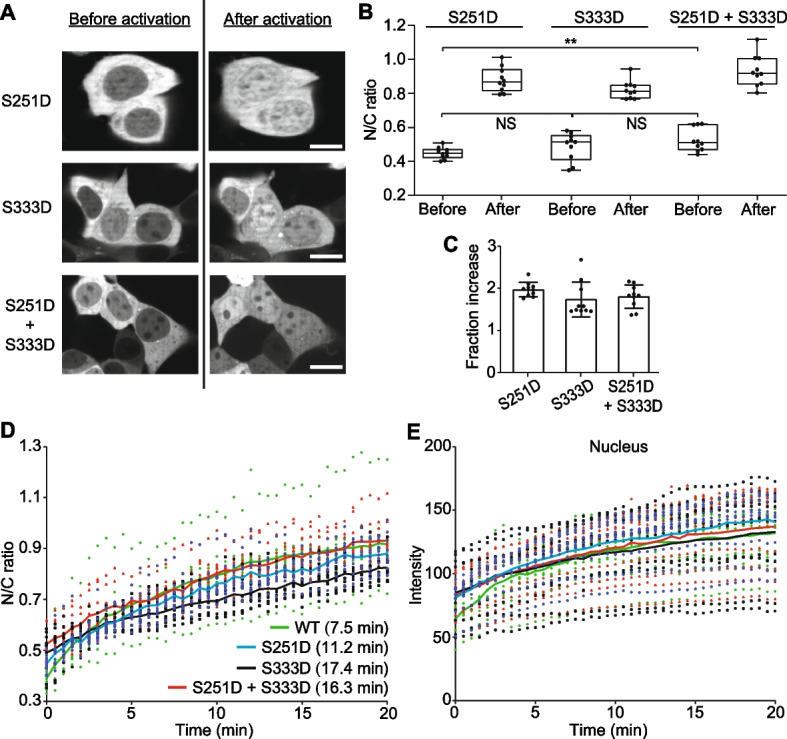
Point mutations of optoYAP at ERK phosphorylation serine residues.** A** Three mutants containing phosphomimetic of ERK target residues at S251, S333 or both S251 and S333. Representative images of HEK293T cells transfected with different mutant optoYAP before and after exposure to pulsatile light activation. Scale bars, 10 µm. **B** Quantification of cells from (**A**), measuring nuclear to cytoplasmic ratio of optoYAP before and after light activation for each mutant. Box plots represent median and 25^th^ to 75^th^ percentiles. Whiskers show minimum and maximum points. NS: not significant, **P < 10^–2^. **C** Fraction increase of N/C ratio after light activation. Bar represents average and error bars represent s.d. **D** Relative N/C ratio over time during 488 nm activation amongst optoYAP mutants. **E** Absolute intensity values of mCherry signal in nuclei over time. Solid lines represent mean, n = 10 cells taken from three independent experiments

Previous studies have shown that the MAPK/ERK pathway is a key regulator of organ regeneration [33, 34], and crosstalk’s with the Hippo-YAP pathway in cardiomyocytes to reactivate cell proliferation [22, 35]. ERBB2 (a member of the epidermal growth factor receptor (EGFR) family) signalling induces a phosphorylation mark on two non-canonical serine residues on YAP by ERK [22]. The activating phosphorylation marks on S251 and S333 oppose the conventional deactivating phosphorylation caused by LATS on S127. Phosphorylation of YAP on the two ERK activating sites appears to be critical for wound healing [22], but the dynamics remain poorly understood. To explore this further, we synthesised three phosphomimetic optoYAP mutants by substituting serine to aspartic acid on either S251 or S333, or both. We did this in HEK293T cells due to their ease of transfection over H9c2 or MKN28 cells. Despite no clear visual differences amongst the three mutants of optoYAP in the dark (Fig. 4A), quantification of the nuclear-cytoplasmic ratio of optoYAP showed that the double mutant had a significantly higher initial nuclear localisation compared to the S251D mutant, but not the S333D mutant (Fig. 4B). The mutants all displayed clear response to light activation (Fig. 4B–D) and higher initial nuclear localisation over wildtype optoYAP prior to light activation (Fig. 4E).

**Fig. 5:**
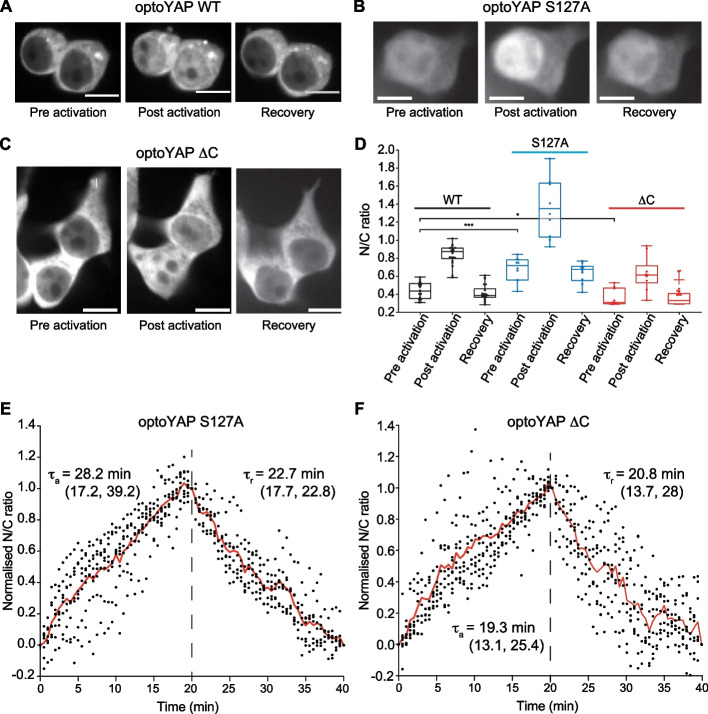
optoYAP variants under light activation. Representative images of mCherry signal in HEK293T cells containing **A** wildtype (WT) optoYAP, **B** optoYAP with a point mutation at S127A, or **C** truncated optoYAP without the C-terminal PDZ-binding motif (optoYAP ΔC). The ‘Pre activation’ panels show cells before exposure to 488 nm light, and after 20 min of pulsatile activation with 488 nm light, the same cells were imaged as ‘Post activation’. Cells were left in the dark for a 20 min and imaged again, under the ‘Recovery’ panel. **D** Quantification of nuclear to cytoplasmic ratio of optoYAP in (**A**–**C**). Box plots represent median and 25th to 75th percentiles. Whiskers show minimum and maximum points. Scale bars, 10 µm. n = 22 cells for (**A**), n = 10 cells for (**B**, **C**), taken from three independent experiments. *P < 0.05, ***P < 10^–3^. **E**, **F** Quantification of activation (τ_a_) and recovery (τ_r_) time constant of **E** S127A mutant and **F** ΔC mutant during light activation and recovery. Red line represents average and vertical dashed line represents time when 488 nm stimulation ceased. Numbers in brackets represent the 95% confidence interval of τ. n = 8 cells

We then looked at canoncial YAP regulatory sites, such as serine 127 and the C-terminal PDZ-binding motif. The phosphorylation mark at S127 generates a 14–3-3 binding site, causing the cytoplasmic sequestration of phosphorylated YAP, accelerating the nuclear export of YAP and inhibiting its co-transcriptional function inside the nucleus [36, 37]. Mutation of serine 127 to alanine has been reported to increase the activity of YAP by preventing LATS phosphorylation [26, 38, 39]. We observed that the S127A mutant (referred to as optoYAP S127A) is nuclear localised even before light activation, contrasting significantly with the wildtype cells (Fig. 5A, B). After light activation, we observed that optoYAP S127A accumulated further within the nucleus, doubling the nuclear to cytoplasmic ratio of optoYAP S127A as compared to the dark state (Fig. 5D). This shows that the optoYAP S127A mutant can exert its predominantly nuclear state but optogenetic activation can intensify this ability and increase optoYAP levels in the nucleus.

Deletion of the PDZ-binding motif on YAP causes complete YAP exclusion from the nucleus [40]. We synthesised a truncated optoYAP without the PDZ-binding motif, which we refer to as optoYAP ΔC. We observed nuclear exclusion of optoYAP ΔC before exposure to light activation (Fig. 5C). Pulsatile light activation can induce nuclear localisation of optoYAP ΔC, nearly doubling the amount of nuclear localised optoYAP ΔC. The optoYAP ΔC construct returns to the cytoplasm after 20 min of recovery in the dark, similar to the wildtype and S127A mutant. Therefore, activation of the optogenetic tool can overcome mutations that drive YAP out of the nucleus.

Since both mutations affect the basal distribution of optoYAP in the dark, we measured the nuclear import and export dynamics of both optoYAP S127A and ΔC mutants to assess the difference in nuclear shuttling (Fig. 5E, F). The optoYAP S127A mutant has a slower activation time constant (τ_a_, 28.2 min) as compared to the ΔC mutant (19.3 min). Similarly, nuclear export is longer in S127A mutant (τ_r_, 22.7 min) than that in ΔC.

## Discussion

Here, we report that optoYAP can accelerate wound healing by promoting cell migration in different cell culture systems. We show that activation of YAP can increase the rate of wound repair by 20% in H9c2 cardiomyocytes. optoYAP, in the absence of endogenous YAP in MKN28 cells, promotes wound healing, accelerating the time taken for closure (Fig. 3A). However, we observed smaller differences in the H9c2 cells between the wildtype and the optoYAP transfected cells (Fig. 2A, B). We further explored the mechanisms that determine the YAP nuclear-cytoplasmic localisation (Figs. 4 and 5). Taken together, our results provide strong evidence that: (i) YAP can play an active role in repair beyond simply proliferation; and (ii) optoYAP can regulate YAP activity, even in the presence of different perturbations to YAP, which may be important for wound repair in a range of cell types.

We saw that optoYAP increases the amount of cell proliferation in serum starved MKN28 *YAP*^*−/−*^ cells, but not to the wildtype levels (Fig. 3B). On the contrary in H9c2 cells, activated optoYAP does not alter the amount of proliferation in transfected cells (Fig. 2C). Light activated optoYAP transfected MKN28 *YAP*^*−/−*^ cells took a shorter time for wound closure as compared to their wildtype counterparts, suggesting that optoYAP promotes cell migration in these wound healing assays. Similarly, in the H9c2 cells, we see that the amount of proliferation events amongst the three conditions (wildtype, dark and light) are comparable (Fig. 2C) but light activated optoYAP took the quickest time for the wound healing assay. Therefore, we believe that both H9c2 and MKN28 cell lines show the same conclusion that light activated optoYAP does not induce cell proliferation in serum-free conditions but promotes cell migration for the wound healing assay. This also suggests a potential broader conclusion: that coordinated cell migration is a dominant driver of wound healing over proliferation, at least in our cell lines.

Multiple signalling pathways regulate YAP independently of Hippo, including the EGFR signalling pathway [41]. Downstream of EGFR lies the Ras/Raf/MEK/ERK transduction axis, where ERK is the final kinase activator that regulates transcription factors. ERK is a key regulator of regeneration and interplay between ERK and YAP has been shown to be drive YAP activation. Nuclear localisation of YAP phosphorylated at both activating serine residues have been observed, though a single phosphorylation mark at either serine residue is sufficient to induce nuclear translocation [42, 43]. The double phosphomimetic mutant was significantly more nuclear localised than the S251D single mutant (Fig. 4B), suggesting that the two serine targets have an additive effect by phosphorylating both serine residues.

Phosphorylated YAP/TAZ triggers its cytoplasmic sequestration [44, 45] or degradation [46], while unphosphorylated YAP/TAZ can translocate into the nucleus and bind to transcription factors TEAD1-4 [25, 47, 48]. This stimulates the expression of a range of genes that typically lead to pro-survival outcomes in the cell [49–51]. Besides the phosphorylation site at S127, another protein domain that regulates the localisation of YAP is the C-terminal PDZ-binding motif [52], a short motif of only five amino acid residues, -FLTWL at the very carboxyl terminal end. This motif allows YAP to bind to proteins containing the PDZ domain such as ZO-2, and a deletion of this motif impairs the nuclear shuttling of YAP through ZO-2 [52]. However, similar to the S127A mutant, truncation of the PDZ-binding motif does not impair the induction of TEAD-mediated transcriptional activity, only affecting its subcellular localisation [39, 40]. The YAP paralogue TAZ also contains a C-terminal PDZ-binding motif, which has been shown to be required for its nuclear localisation [45]. Therefore, the PDZ-binding motif appears to be a conserved sequence that is integral to the function of YAP/TAZ as transcriptional co-regulators.

Although the basal levels differ for the variants, nuclear localisation doubles for all optoYAP variants in the light and decreases back to their respective basal levels after recovery in the dark (Fig. 5D). This shows that the optogenetic domain can promote further nuclear translocation despite mutations that already boosts (S127A) or inhibit (ΔC) nuclear localisation. Furthermore, since the optoYAP S127A mutant is already nuclear localised in the dark, nuclear import rates during light activation are slower than that of the ΔC mutant (Fig. 5E, F). The mutation on S127A impedes optoYAP from translocating out of the nucleus, therefore the time taken for nuclear export is also longer as compared to the ΔC mutant.

Finally, we note that several optogenetic YAP constructs have been published recently, with different YAP isoforms and activation protocols. Illes et al. first published a tool to control the subcellular localisation of YAP by tagging it with an optogenetic NLS, shuttling YAP between the cytoplasm and the nucleus [53]. The authors used the human YAP1 gene without specifying the details of the splice isoform but demonstrated that activated YAP increases proliferation in 2D and 3D cultures and induces invasion in cancer spheroids [53]. Dowbaj et al*.* presented optogenetic YAP1, using the human YAP1-2γ isoform [54], which contains exon 6 and two WW domains [55], tethering optogenetic YAP and TAZ to the mitochondria. Their tool allows the study of protein dynamics by describing the rate of nuclear entry and exit but does not demonstrate downstream functionality of nuclear YAP [54]. In our previous work, we reported a human YAP1-1δ optogenetic tool that translocates between the cytoplasm and nucleus, demonstrating functional outputs in vitro and in vivo [21]. Most recently, Meyer et al*.* have developed a mouse YAP1 tool. They are able to regulate the levels of pluripotency factors Oct4 and Nanog, and hence control the renewal of a naïve stem cell state or preferential differentiation into the mesendodermal state [56]. The various YAP isoforms signal differently, where those without exon 6, alpha and beta, can form heterodimers with TAZ [57]. Additionally, different isoforms have unique and common functions and activate distinct downstream transcriptional programmes [58]. Taken together, the isoforms of YAP matter when describing optogenetic constructs, given that each isoform can have unique downstream effects.

The role of YAP in wound healing has been investigated previously, where nuclear localisation of YAP is observed during healing [59, 60]. We demonstrate that light-activated optoYAP can accelerate wound healing without an increase in proliferation, but rather in cell migration. It has been shown that H9c2 cells respond to substrate stiffness and topography, promoting cell differentiation on a modified extracellular matrix (ECM) synthesized from fibroblast-derived matrix [61]. With our optogenetic tool, it will be interesting to investigate cell migration in an environment that mimics physiological conditions by seeding cells on permissive ECM, or a stiffened matrix that imitates cholesterol plaques, and tracking wound healing under such conditions, which will benefit future studies on regeneration.

## Supplementary Information

**Additional file 1. **Schematic of different YAP splicing isoforms from human or murine origins. Human YAP1-1δ isoform used in this study contains only 1 WW domain as compared to the mouse YAP1-2α isoform that Aharonov et al. used in their publication [22]. There are also splicing and protein size differences in both human and mouse YAP proteins, which explains why the serine residues are not at the same position in both proteins.

**Additional file 2. **Sequences of different YAP isoforms from human or murine origins. Top panel shows human YAP1-1δ isoform used in this study and bottom panel shows mouse YAP1-2δ isoform used in Aharonov et al. [22]. Sequences highlighted in bold show conserved portions of the protein in both orthologs, with the regulatory serine residues highlighted in red within the conserved regions. The sequence explains the discrepancy in the absolute position of both serine residues derived from different YAP isoforms.

## Data Availability

The datasets used and/or analysed during this study are available from the corresponding author on reasonable request.

## Abbreviations

cDNA: Complementary DNA
Ct: Threshold cycle
DMEM: Dulbecco’s modified Eagle’s medium
ECM: Extracellular matrix
EGFR: Epidermal growth factor receptor
FBS: Fetal bovine serum
NA: Numerical aperture
NEAA: Non-essential amino acids
NFW: Nuclease-free water
NLS: Nuclear localisation sequence
ROI: Region of interest
RT-qPCR: Quantitative real-time polymerase chain reaction
SDM: Site-directed mutagenesis
STR: Short tandem repeat
WT: Wildtype
