# Supplementary figures and images for: Optogenetic control of YAP can enhance the rate of wound healing
Source: Cell Mol Biol Lett. 2023 May 11;28:39. doi: 10.1186/s11658-023-00446-9 (PMC10176910; doi:10.1186/s11658-023-00446-9)

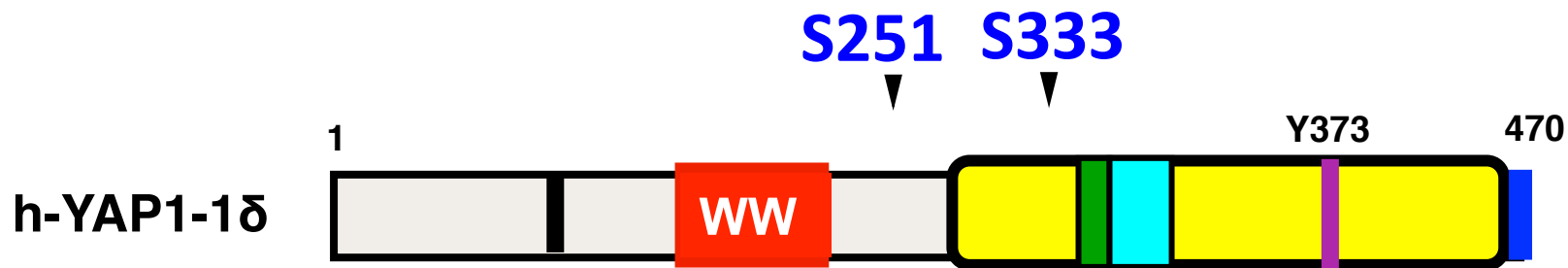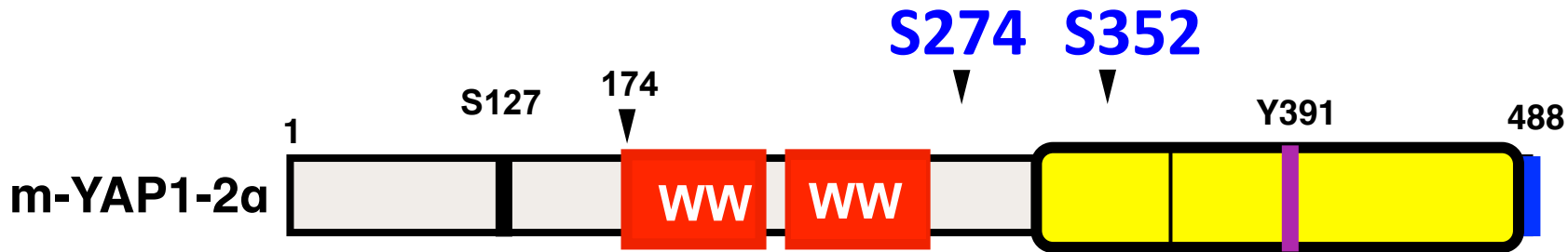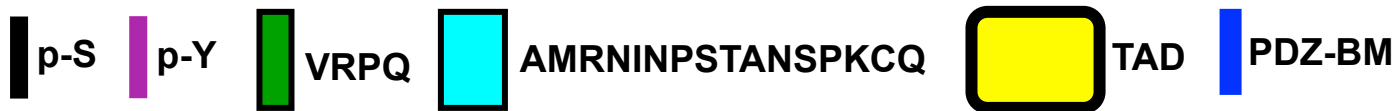

Supplement: Supplementary file 1 — Additional file 1. Schematic of different YAP splicing isoforms from human or murine origins. Human YAP1-1δ isoform used in this study contains only 1 WW domain as compared to the mouse YAP1-2α isoform that Aharonov et al. used in their publication [22]. There are also splicing and protein size differences in both human and mouse YAP proteins, which explains why the serine residues are not at the same position in both proteins. [file 11658_2023_446_MOESM1_ESM.pdf]
