# Supplementary material for: Optogenetic control of YAP can enhance the rate of wound healing
Source: Cell Mol Biol Lett. 2023 May 11;28:39. doi: 10.1186/s11658-023-00446-9 (PMC10176910; doi:10.1186/s11658-023-00446-9)
Supplement: Supplementary file 2 — Additional file 2. Sequences of different YAP isoforms from human or murine origins. Top panel shows human YAP1-1δ isoform used in this study and bottom panel shows mouse YAP1-2δ isoform used in Aharonov et al. [22]. Sequences highlighted in bold show conserved portions of the protein in both orthologs, with the regulatory serine residues highlighted in red within the conserved regions. The sequence explains the discrepancy in the absolute position of both serine residues derived from different YAP isoforms. [file 11658_2023_446_MOESM2_ESM.pdf]

The S residues S251 and S333 are shown in red font in 470 amino acid long human YAP 1-1 delta isoform.

mdpgqqpppqpapqgqgqppsqppqgqgppsgpgqpapaatqaapqappaghqivhvrgdsetdlealfna
vmnpktanvpqtvpmrlrklpdsffkppepkshsrqastdagtagaltpqhvrahsspaslqlgavspgtl
tptgvvsgpaatptaqhlrqssfeipddvplpagwemaktssgqryflnhidqtttwqdprkamlsqmnvt
aptsppvqqnmmnsasamnqrisqsapvkqppp**lapq**__**s**__**pq**ggvmggsnsnqqqmrlqqlqmekerlrlkq
qellrqvrpqamrninpstanspkcqelalrsqlptleqdggtqnp**vs**__**s**__**p**gmsqelrtmttnssdpflnsg
tyhsrdestdsglsmssysvprtpddflnsvdemdtgdtinqstlpsqqnrfpdyleaipgtnvdlgtleg
dgmniegeelmpslqealssdilndmesvlaatkldkesfltwl

The S residues S274 and S352 are shown in red font in 488 amino acid long mouse YAP 1-2 alpha isoform.

MEPAQQPPPQPAPQGPAPPSVSPAGTPAAPPAPPAGHQVVHVRGDSETDLEALFNAVMNPKTANVPQTVP
MRLRKLPDSFFKPPEPKSHSRQASTDAGTAGALTPQHVRAHSSPASLQLGAVSPGTLTASGVVSGPAAAP
AAQHLRQSSFEIPDDVPLPAGWEMAKTSSGQRYFLNHNDQTTTWQDPRKAMLSQLNVPAPASPAVPQTLM
NSASGPLPDGWEQAMTQDGEVYYINHKNKTTSWLDPRLDPRFAMNQRITQSAPVKQPPP**LAPQ**__**S**__**P**QGGVL
GGGSSNQQQQIQLQQLQMEKERLRLKQQELFRQAIRNINPSTANAPKCQELALRSQLPTLEQDGGTPNA**V**
S__**S**__**P**GMSQELRTMTTNSSDPFLNSGTYHSRDESTDSGLSMSSYSIPRTPDDFLNSVDEMDTGDTISQSTLP
SQQSRFPDYLEALPGTNVDLGTLEGDAMNIEGEELMPSLQEALSSEILDVESVLAATKLDKESFLTWL
